# Optogenetic Patterning of Whisker-Barrel Cortical System in Transgenic Rat Expressing Channelrhodopsin-2

**DOI:** 10.1371/journal.pone.0093706

**Published:** 2014-04-02

**Authors:** Tatsuya Honjoh, Zhi-Gang Ji, Yukinobu Yokoyama, Akira Sumiyoshi, Yuma Shibuya, Yoshiya Matsuzaka, Ryuta Kawashima, Hajime Mushiake, Toru Ishizuka, Hiromu Yawo

**Affiliations:** 1 Department of Developmental Biology and Neuroscience, Tohoku University Graduate School of Life Sciences, Sendai, Japan; 2 Japan Science and Technology Agency (JST), Core Research of Evolutional Science & Technology (CREST), Tokyo, Japan; 3 Institute of Development, Aging and Cancer, Tohoku University, Sendai, Japan; 4 Department of Physiology, Tohoku University, Graduate School of Medicine, Sendai, Japan; 5 Center for Neuroscience, Tohoku University Graduate School of Medicine, Sendai, Japan; University Paris 6, France

## Abstract

The rodent whisker-barrel system has been an ideal model for studying somatosensory representations in the cortex. However, it remains a challenge to experimentally stimulate whiskers with a given pattern under spatiotemporal precision. Recently the optogenetic manipulation of neuronal activity has made possible the analysis of the neuronal network with precise spatiotemporal resolution. Here we identified the selective expression of channelrhodopsin-2 (ChR2), an algal light-driven cation channel, in the large mechanoreceptive neurons in the trigeminal ganglion (TG) as well as their peripheral nerve endings innervating the whisker follicles of a transgenic rat. The spatiotemporal pattern of whisker irradiation thus produced a barrel-cortical response with a specific spatiotemporal pattern as evidenced by electrophysiological and functional MRI (fMRI) studies. Our methods of generating an optogenetic tactile pattern (OTP) can be expected to facilitate studies on how the spatiotemporal pattern of touch is represented in the somatosensory cortex, as Hubel and Wiesel did in the visual cortex.

## Introduction

Animals perceive the external world through sensory systems consisting of the peripheral sensory organs, sensory nerves and the central nervous system (CNS). For example, rodents use their whiskers/vibrissae to collect information about objects: position, size, shape and texture [1]–[3]. The principal whiskers/vibrissae are two-dimensionally arrayed on the snout and innervated by the maxillary branch of the trigeminal nerve. The trigeminal ganglion (TG) neurons innervating the whisker follicles transmit the afferent signal to the primary somatosensory cortex called “barrels” through the brainstem and thalamus. The layer IV projection is somatotopically patterned so as to map the whisker arrangement of the contralateral side, although some thalamo-cortical projections have broad receptive fields. Therefore, the rodent whisker-barrel system has been an ideal model for studying somatosensory representations in the cortex.

Previously, we generated several transgenic lines of rat which express channelrhodopsin-2 (ChR2), an algal light-driven cation channel, under the control of thy1.2 promoter [4]. In one of them, W-TChR2V4, ChR2 was exclusively expressed in a subpopulation of large mechanoreceptive neurons in the dorsal root ganglion (DRG) but not in the small-sized neurons, which are involved in nociception [5]. Furthermore, ChR2 was also expressed in their peripheral nerve endings such as those innervating Merkel corpuscles and Meissner corpuscles, which are involved in the sense of touch. When the plantar skin was irradiated by blue light, these transgenic rats showed a touch-sensitive behavior. In the present study, we identified that ChR2 is also expressed in the large mechanoreceptive neurons but not in the nociceptive ones in the TG. As the whisker follicles were densely innervated by the ChR2-positive mechanoreceptive nerve endings, the blue light irradiation of them evoked electrical and fMRI responses in the barrel field of the contralateral somatosensory cortex. It is suggested that the patterned cortical activity is induced by the direct optogenetic stimulation of the peripheral nerve endings of the whisker follicles in a given spatiotemporal pattern. The preliminary report has been published elsewhere [6].

## Results

### ChR2 expression in the primary sensory neurons

**Figure 1:**
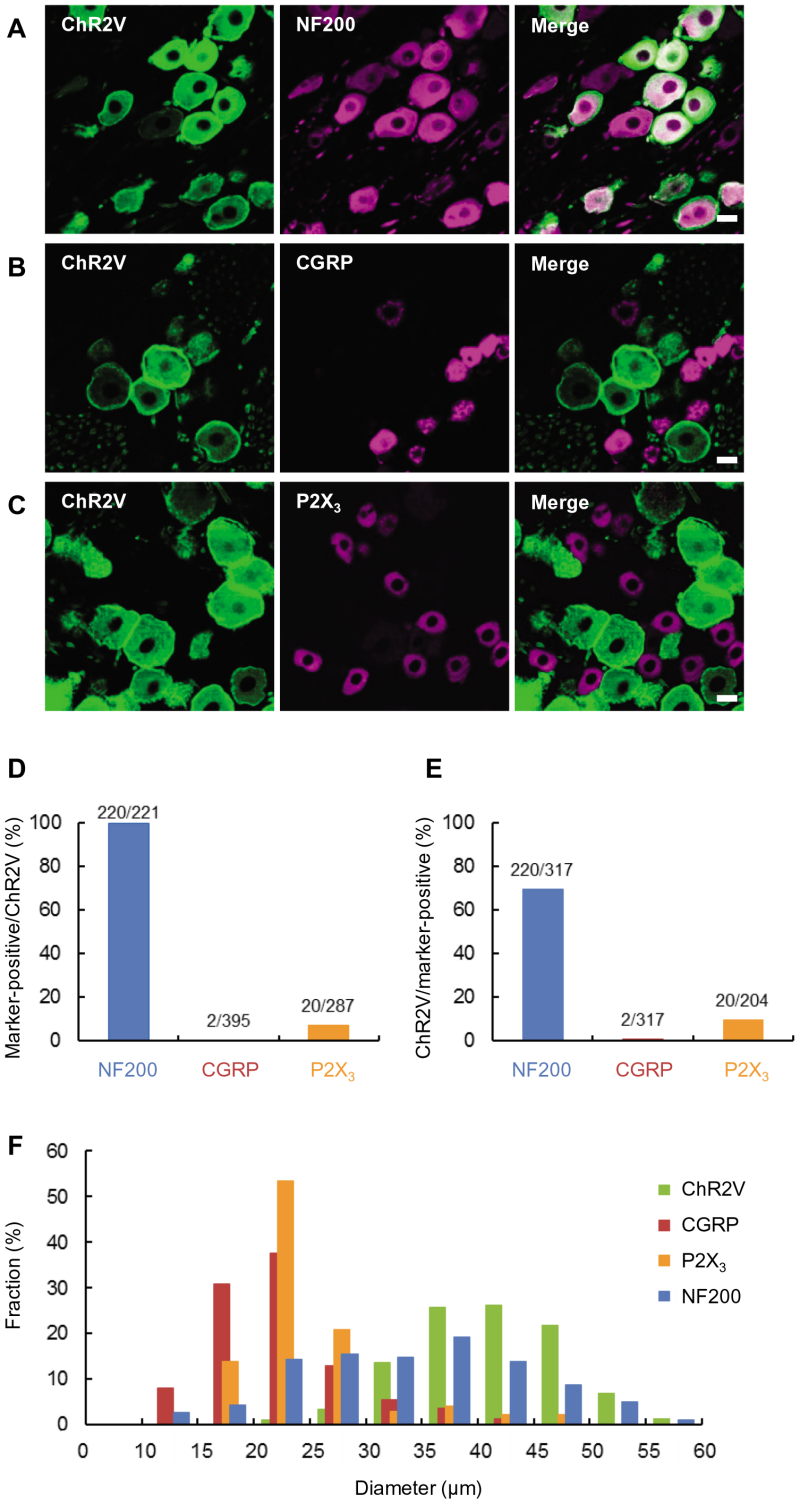
Distribution of channelrhodopsin 2-Venus conjugates (ChR2V) in the trigeminal ganglion (TG) neurons. **A-C**, Immunohistochemical identification of ChR2V-positive (ChR2V+) neurons using cell-type specific markers, NF200 (A), CGRP (B) and P2X_3_ (C). Scale bars indicate 20 μm. **D**, Probability of the co-expression of each marker, NF200, CGRP or P2X_3_, in the ChR2V+ neurons. **E**, Probability of the co-expression of ChR2V in the neurons positive for each marker, NF200, CGRP or P2X_3_. **F**, The average diameters of the TG neurons were compared among four groups positive for ChR2V (green), NF200 (blue), CGRP (red) and P2X_3_ (orange).

The distribution of ChR2-Venus conjugates (ChR2V) was immunohistochemically identified using the W-TChR2V4 line. As shown in Fig. 1A, the ChR2V-positive (ChR2V+) TG neurons usually co-expressed NF200 (220/221 neurons, 99.5%, Fig. 1D), one of the markers of myelinated neurons, although not all NF200-positive neurons were ChR2V+ (220/317, 69%, Fig. 1E). On the other hand, almost negligible numbers of the ChR2V+ TG neurons co-expressed calcitonin gene-related peptide (CGRP) (2/395 neurons, 0.5%, Fig. 1B and 1D). A small number of the ChR2+ TG neurons co-expressed P2X_3_ (20/287 neurons, 7.0%) (Fig. 1C and 1D).

The size of each TG neuron was evaluated by its average diameter, as summarized in Fig. 1F. The ChR2V+ TG neurons (diameter, 41±0.38 μm, n = 302) were clearly discriminated in terms of size from the CGRP-positive TG neurons (diameter, 22±0.47 μm, n = 162), with statistical significance (P << 10^−10^, two-tailed *t*-test). Their size distribution was also different from that of the P2X_3_-positive TG neurons (diameter, 25±0.49 μm, n = 172; P << 10^−10^, two-tailed *t*-test), although some P2X_3_-positive neurons had diameters between 35 and 50 μm (15/172 neurons, 8.7%). On the other hand, there was no significant difference in size between the CGRP- and the P2X_3_-positive groups. The size of NF200-positive neurons were widely distributed from 10–60 μm (diameter, 34±0.75 μm, n = 181).

**Figure 2:**
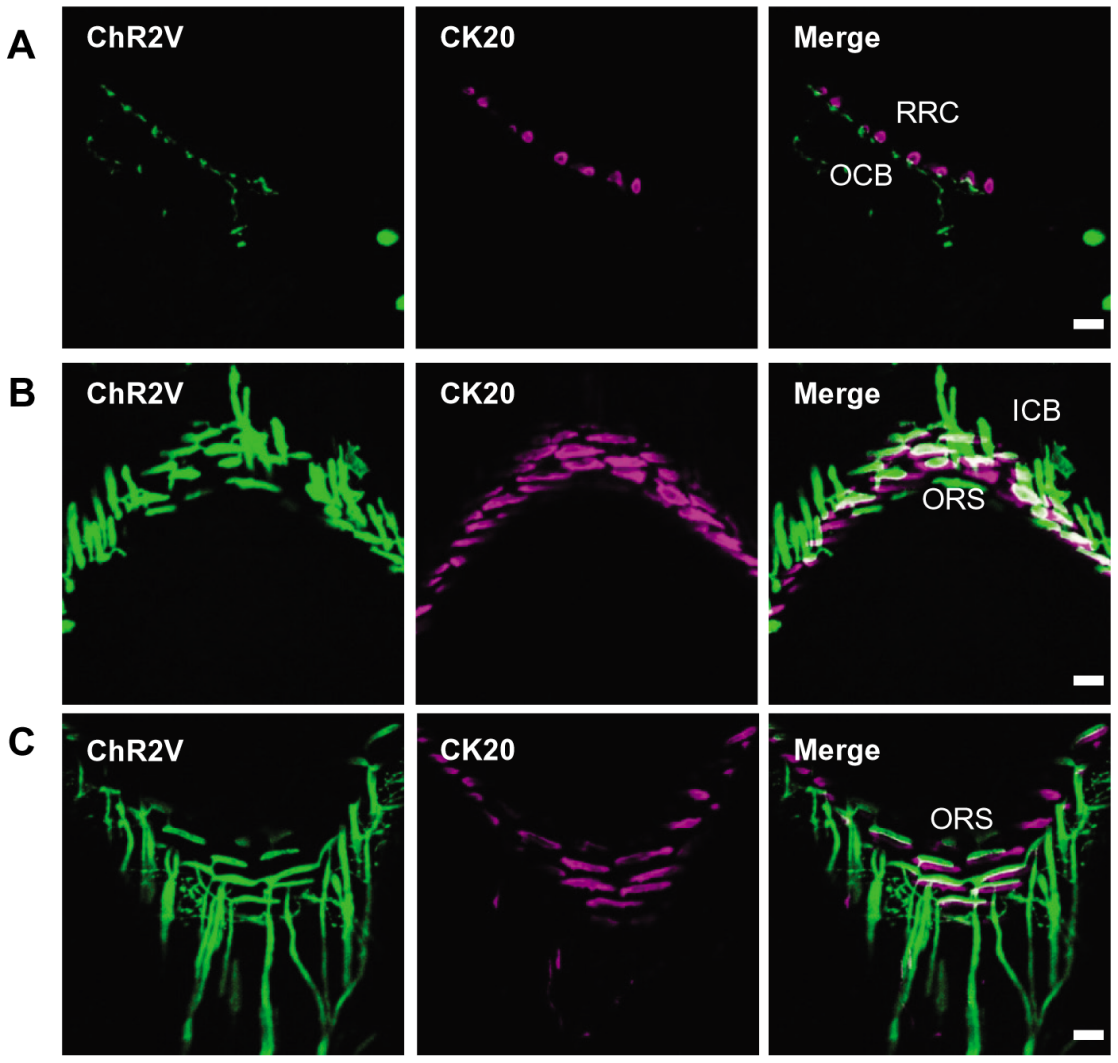
ChR2V-positive peripheral nerve endings around whisker follicles. **A**, The ChR2V-immunoreactive nerve endings (green) at superficial layer around the whisker follicle where they were associated with CK20-positive Merkel cells (magenta). **B**, The ChR2V-positive nerve endings at middle layer. **C**, The ChR2V-positive nerve endings at deep layer. Abbreviations: RRC, rete ridge collar; OCB, ourter conical body; ICB, inner conical body; ORS, outer root sheath; MS, mesenchymal sheath. Scale bars indicate 20 μm.

Each whisker follicle is innervated by a single, relatively large deep vibrissal nerve (DVN), and several smaller superficial vibrissal nerves (SVNs) [7]. To test whether these nerves expressed ChR2V, we examined the follicle-sinus complexes (FSCs) of the whiskers at three levels: (1) the superficial layer consisting of the rete ridge collar (RRC) and outer conical body (OCB); (2) the middle layer including the inner conical body (ICB); (3) the deep layer surrounded by the ring sinus (RS). The superficial layer was frequently innervated by fine ChR2V+ nerves with varicosities that were opposed to the CK20-positive Merkel cells (Fig. 2A). In the middle layer, the ChR2V+ nerves innervated circumferentially around the outer root sheath (ORS) of the whisker and formed lanceolate endings, which were closely associated with CK20-positive Merkel cells (Fig. 2B). The deep layer was richly innervated by ChR2V+ nerves, which had lanceolate or club-like endings associated with CK20-positive Merkel cells (Fig. 2C).

### Light-sensitivity of the ChR2-expressing TG neurons

**Figure 3:**
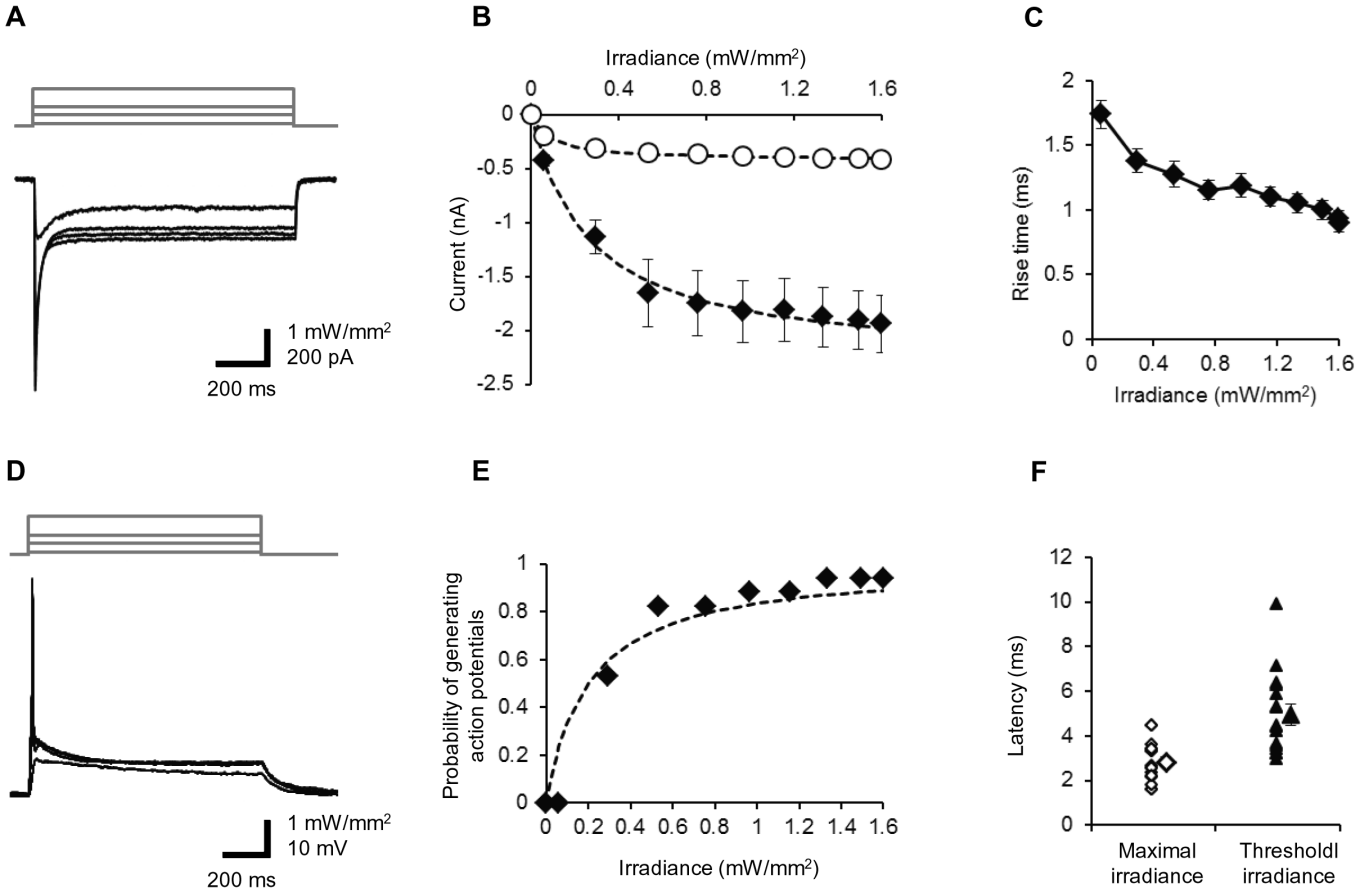
Optical responses of the ChR2V+ TG neurons. **A**, Representative records of photocurrents (bottom) evoked by blue LED light of variable strength (top) under voltage clamp. **B**, The peak (closed diamonds) and the steady-state (open circles) photocurrent amplitudes as functions of the light power density. Each point with bar indicates mean ± SEM and is fitted to a Michaelis-Menten-type kinetics, *y* = *I_max_ x* / (*x* + *K_m_*); *I_max_* = −2.3 nA (peak) and 0.42 nA (steady state), respectively, and *K_m_* = 0.26 mWmm^−2^ (peak) and 0.085 mWmm^−2^ (steady state), respectively. **C**, The time between the onset of LED pulse and the minimal rate of rise of ChR2 photocurrent was measured as the rise time and plotted as a function of irradiance (mean ± SEM, n = 29). **D**, Representative records of the neuronal membrane potential evoked by blue LED pulses (200 ms) of variable strength under current clamp. The resting membrane potential, −56 mV. **E**, The probability of generating action potentials as a function of the irradiance (n = 14). Each point with bar indicates mean ± SEM and is fitted to a Michaelis-Menten-type relationship, *y* = *x* / (*x* + *K_m_*) where *K_m_* = 0.20 mWmm^−2^. **F**, The time between the onset of the LED pulse and the maximal rate of rise of action potential was measured as the action potential rise time and plotted for both responses to the maximal irradiance (open diamonds, n = 16) and the threshold irradiance (closed triangles, n = 16). The larger symbols represent mean ± SEM.

Since ChR2V was expressed in TG neurons, we could also expect that the ChR2V-expressing neurons would be responsive to light by generating either a photocurrent or action potentials. To this end, the TG neurons were cultured and the ChR2V expression was identified by the presence of Venus fluorescence. Under whole-cell voltage clamp, blue LED light pulse evoked a photocurrent in every ChR2V-expressing neuron (15/15 neurons). Both the peak and the steady-state photocurrents were dependent on the irradiance (Fig. 3A and 3B). The peak current ranged between 0.65 and 5.0 nA (n = 15) at the maximal irradiance, although unclamped currents from escaped action potentials were frequently observed. The photocurrent rise time was also dependent on the irradiance and was less than 1 ms at its maximum (Fig. 3C). Under the current clamp, the blue LED light pulse (200 ms) evoked rapid membrane depolarization in an intensity-dependent manner and, eventually, only one action potential (Fig. 3D) while the resting potential was −70±1.6 mV (n = 17), the input resistance was 1240±93 MΩ (n = 17) and the membrane time constant was 14±1.7 ms (n = 17). The size of the action potential varied from cell to cell (range, 39–87 mV, n = 17) with threshold irradiances of 0.29–1.3 mWmm^−2^. Thus, as shown in Fig. 3E, the probability of generating action potentials was dependent on the irradiance and 53% (9/17 neurons) fired action potentials at 0.29 mWmm^−2^, 94% (16/17 neurons) at 1.3 mWmm^−2^ and 1/18 neurons, which had the smallest input resistance (550 MΩ), did not fire even at the maximal irradiance (1.6 mWmm^−2^). The minimal irradiance that was necessary to generate an action potential (threshold irradiance) had a weak positive correlation to the input conductance, the reciprocal of input resistance (r = 0.77, n = 16) (Figure S1). The latency to evoke an action potential was 1.6–4.5 ms (mean, 2.8±0.23 ms, n  = 16) from the LED pulse onset of the maximal irradiance (Fig. 3F). This time was on average 5.0±0.46 ms (range, 2.9–9.9 ms, n = 16) at the threshold irradiance.

### Cortical responses

**Figure 4:**
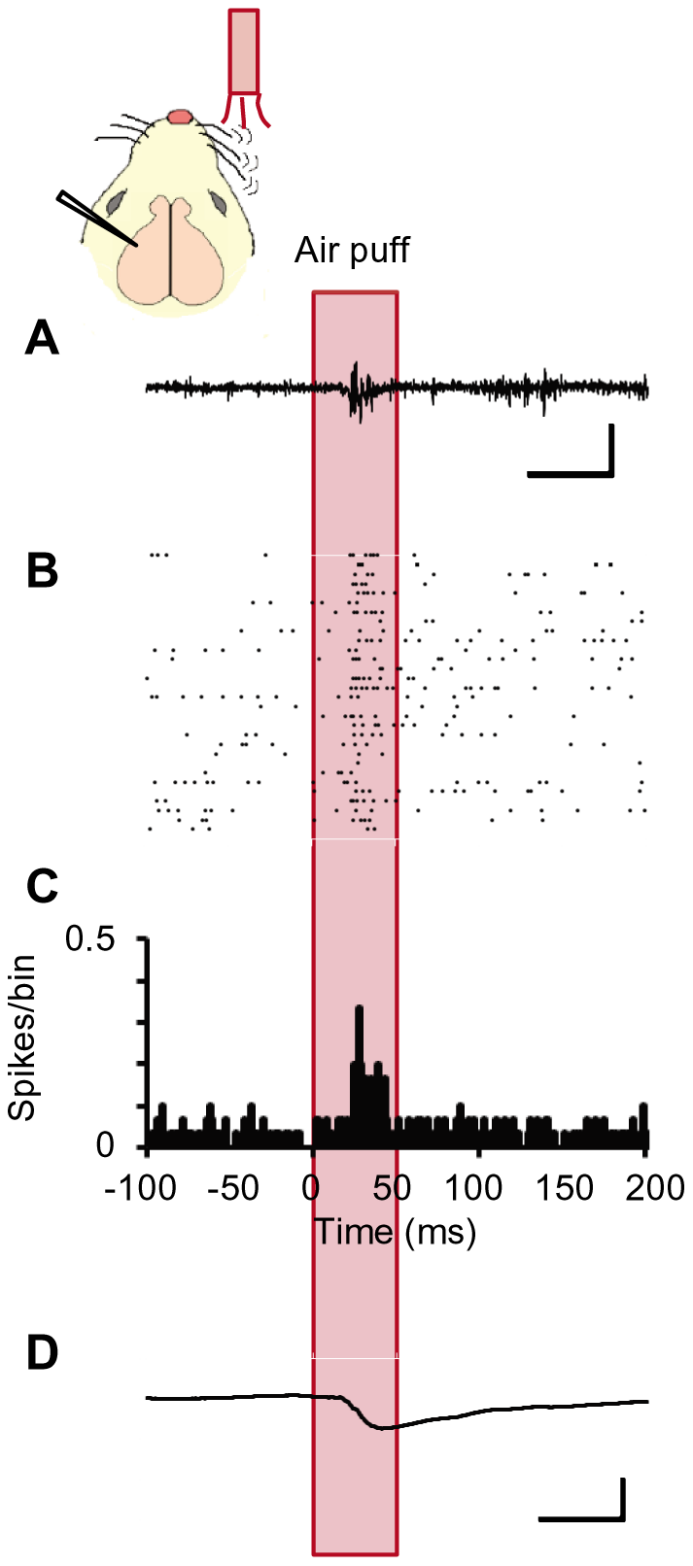
Barrel cortical activities evoked by whisker mechano-stimulation. **A-D**, A sample MUA recording trace (A), raster plots of spikes (B), peristimulus-time histograms (PSTHs, bin width  = 1 ms) of spikes (C) and the averaged LFP record (D) in response to the air puff (duration, 50 ms) to the contralateral whiskers. Scales, 50 ms and 100 μV (A) or 0.5 mV (D).

A tungsten microelectrode was stereotaxically inserted into one barrel in the left cortex and the MUA (multi-unit activity) or LFP (local field potential) of a ChR2V+ rat was recorded under anesthesia while the right whiskers were stimulated by an air puff (Fig. 4). The mechanical stimulation robustly evoked a burst of MUA responses and a negative deflection of LFP in the barrel cortex. The latency to reach the minimal differential of LFP was 21±1.0 ms.

**Figure 5:**
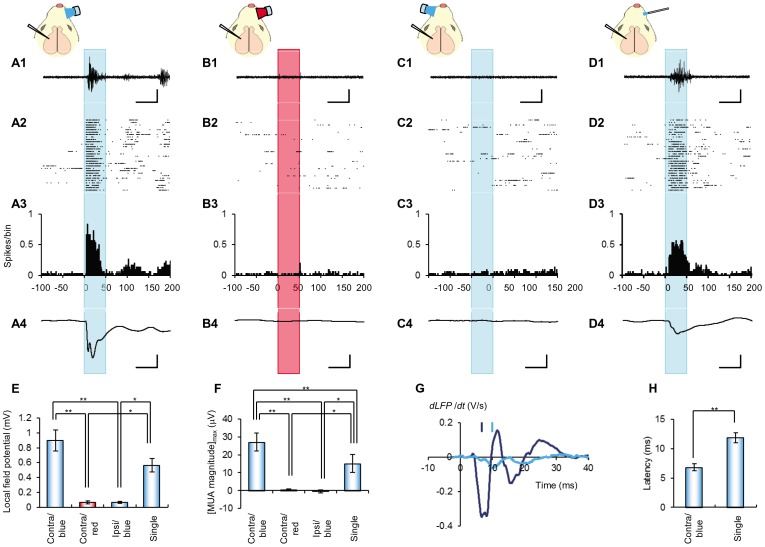
Barrel cortical activities evoked by whisker photostimulation. **A-D**, Sample MUA recording traces (A1-D1), raster plots of spikes (A2-D2), peristimulus-time histograms (PSTHs, bin width = 1 ms) of spikes (A3-D3) and averaged LFP records (A4-D4) during blue irradiation to the contralateral whisker pad (A1-4), red irradiation to the contralateral whisker pad (B1-4), blue irradiation to the ipsilateral whisker pad (C1-4) or fiber-coupled irradiation of blue light to the D3 whisker follicle (D1-4). Scales, 50 ms and 100 μV (A1-D1) or 0.5 mV (A4-D4). **E**, Mean ± SEM of peak amplitudes of averaged LFP for each test; whisker pad irradiations (contralateral blue, contralateral red or ipsilateral blue, n = 7), single whisker irradiation (n = 5). **F**, Mean ± SEM of the maximum value of averaged MUA magnitude for each test. **G**, Sample differential of LFP (*dLFP/dt*) in response to the whisker pad irradiation (A4) and the single whisker irradiation (D4). Each bar indicates the time to reach the minimal *dLFP/dt*. **H**, Summary of LFP latency; whisker pad irradiation (n = 7) and single whisker irradiation (n = 5). The statistical significance was evaluated using paired *t-*test for E, F, and H; *, p<0.05 and **, p<0.005.

Although the blue LED light (470 nm) was absorbed by the whisker-pad skin, 10±1.5% (n = 5) of the energy passed through the epithelium after removing the whiskers and fur. Therefore, we could well expect that the blue light on the whisker follicles should also evoke cortical responses in this transgenic rat (see Discussion). To test this, the snout of the ChR2V+ rat was irradiated by a short pulse of blue or red LED flash at the whisker pad. The duration of LED flash was fixed to 50 ms as the action potentials were usually evoked within 10 ms from the onset of the flash (Fig. 3F and Fig. S2). Indeed, the blue LED flashes robustly evoked cortical responses when they were given to the contralateral (right) whisker pad (Fig. 5A1-4), whereas the red LED flashes or the blue LED flashes to the ipsilateral (left) whisker pad did not (Fig. 5B1-4 and 5C1-4). Then, each whisker follicle was focally irradiated by the fiber-coupled LED in a one-by-one manner to find the place where the light flash evoked the maximal LFP response (Fig. 5D4). To evaluate the effectiveness, the amplitude of the averaged LFP was measured for each test (Fig. 5E). The LFP response to red LED irradiation or to the ipsilateral blue LED irradiation was negligible, as expected. The LFP response to single-whisker irradiation was small but robust and was insignificantly different from that by whisker pad irradiation. To evaluate the MUA magnitude, the standard deviation for each 5 ms-bin of MUA (SD_MUA_) was calculated for each test, then three times of the minimum SD_MUA_ (SD_min_) was subtracted (Figure S3). The MUA magnitude was averaged over time for each test, then the maximal value, [MUA magnitude]_max_ was compard as shown in Fig. 5F. This value was almost negligible by the red LED irradiation or by the ipsilateral blue LED irradiation with significant difference to that by the contralateral blue LED irradiation. The [MUA magnitude]_max_ by the whisker-pad irradiation was also significantly greater than that by the single-whisker irradiation (Fig. 5F).

As shown in Fig. 5A1-4 and 5D1-4, both the MUA and LFP responses were initiated with some delay from the onset of irradiation. The latency to reach the minimal differential of LFP was 6.8±0.60 ms in the case of whisker-pad irradiation (Fig. 5G). It was significantly smaller than that of single-whisker irradiation (11.9±0.86 ms) (Fig. 5H).

**Figure 6:**
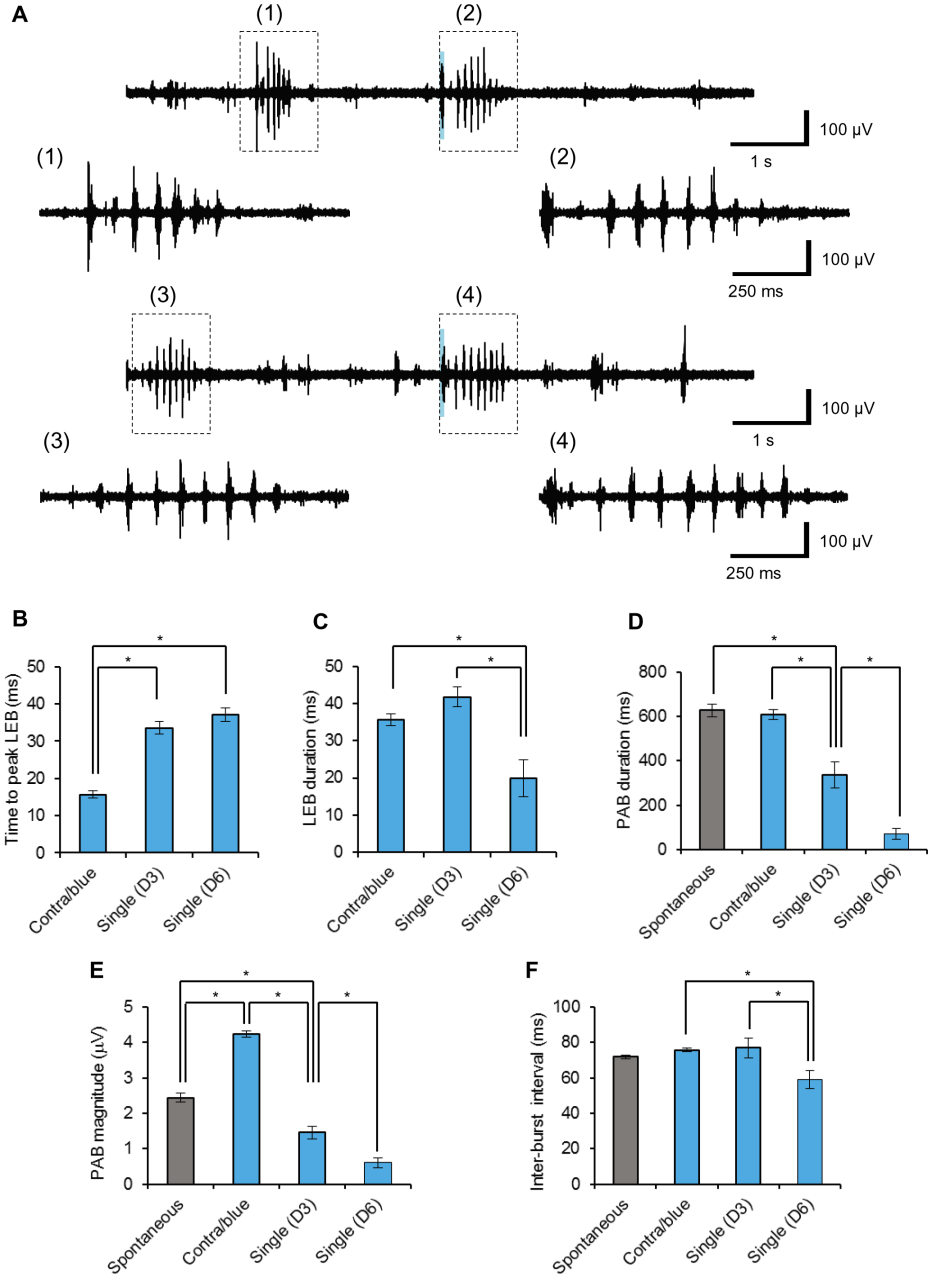
Cortical bursts evoked by whisker photostimulation. **A**, Sample records of MUA bursts in response to the whisker pad irradiation (trace 1) or the D3 single whisker irradiation (trace 2). (1)–(4), The expanded traces shown in each record; (1) and (3), the spontaneous bursts, (2) and (4), light-evoked bursts (LEBs) and afterbursts (PABs). **B-F**, Summary of SD_MUA_ analysis (Figure 3S): the time to reach the peak LEB (B), the LEB duration a (C), the PAB duration (D), the PAB magnitude (E) and the inter-burst interval (F). In B-F, statistical significance was evaluated using one-way ANOVA; *, p<0.05.

The whisker-pad irradiation robustly evoked an immediate burst in the cortical response (light-evoked burst, LEB) of MUA and was followed by a relative quiescence and periodic afterbursts (PAB, Fig. 6A-box 2, 30/30 trials). In this typical experiment the D3 single-whisker irradiation, which evoked the maximal LFP in the same recording place, also robustly evoked the LEB (30/30 trials) and was accompanied by PAB (Fig. 6A-box 4, 24/30 trials). On the other hand, the D6 irradiation induced the LEB in 22/30 trials and PAB in 12/30 trials in the same recording place. The time to peak LEB from the onset of the light pulse (Figure S3) was 16±1.0 ms by the whisker-pad irradiation, a value that was significantly smaller than that by D3 (34±1.8 ms) or D6 irradiation (37±1.8 ms) (Fig. 6B). The LEB duration above 3 times the SD_min_ (Figure S3) was 36±1.6 ms by the whisker-pad irradiation, 42±2.6 ms by the D3 irradiation, but 20±4.9 ms by the D6 irradiation with significant differences (Fig. 6C). These responses were also discriminated by the PAB duration, the duration above three times the SD_min_ (Figure S3), with significant differences (Fig. 6D); the whisker-pad irradiation (610±22 ms, n = 30) > the D3 irradiation (340±57 ms, n = 24) > D6 irradiation (70±24 ms, n = 12). Similar periodic bursts of MUA often occurred spontaneously as shown in Fig. 6A-box 1 and 6A-box 3. The duration of spontaneous bursts was 630±27 ms (n = 30), which was similar to that evoked by the whisker-pad irradiation but significantly larger than that evoked by the D3/D6 irradiation (Fig. 6D). Otherwise the value, the MUA magnitude (SD_MUA_ minus 3 times the SD_min_)_,_ was averaged for each PAB duration and was adopted as the PAB magnitude. The magnitude of spontaneous bursts was also evaluated in a similar manner. As shown in Fig. 6E, the PAB magnitude was dependent on the irradiation pattern with significant differences; the whisker-pad irradiation (4.2±0.094 μV, n = 30) > the spontaneous bursts (2.4±0.12 μV, n = 30) > the D3 irradiation (1.5±0.18 μV, n = 24) > D6 irradiation (0.60±0.14 μV, n = 12). The inter-burst interval was measured between one peak SD_MUA_ and the next and averaged for a PAB (Figure S3). The values were similar in the whisker-pad irradiation, the D3 irradiation and the spontaneous burst, but the value was significantly smaller in the case of D6 irradiation (Fig. 6F).

**Figure 7:**
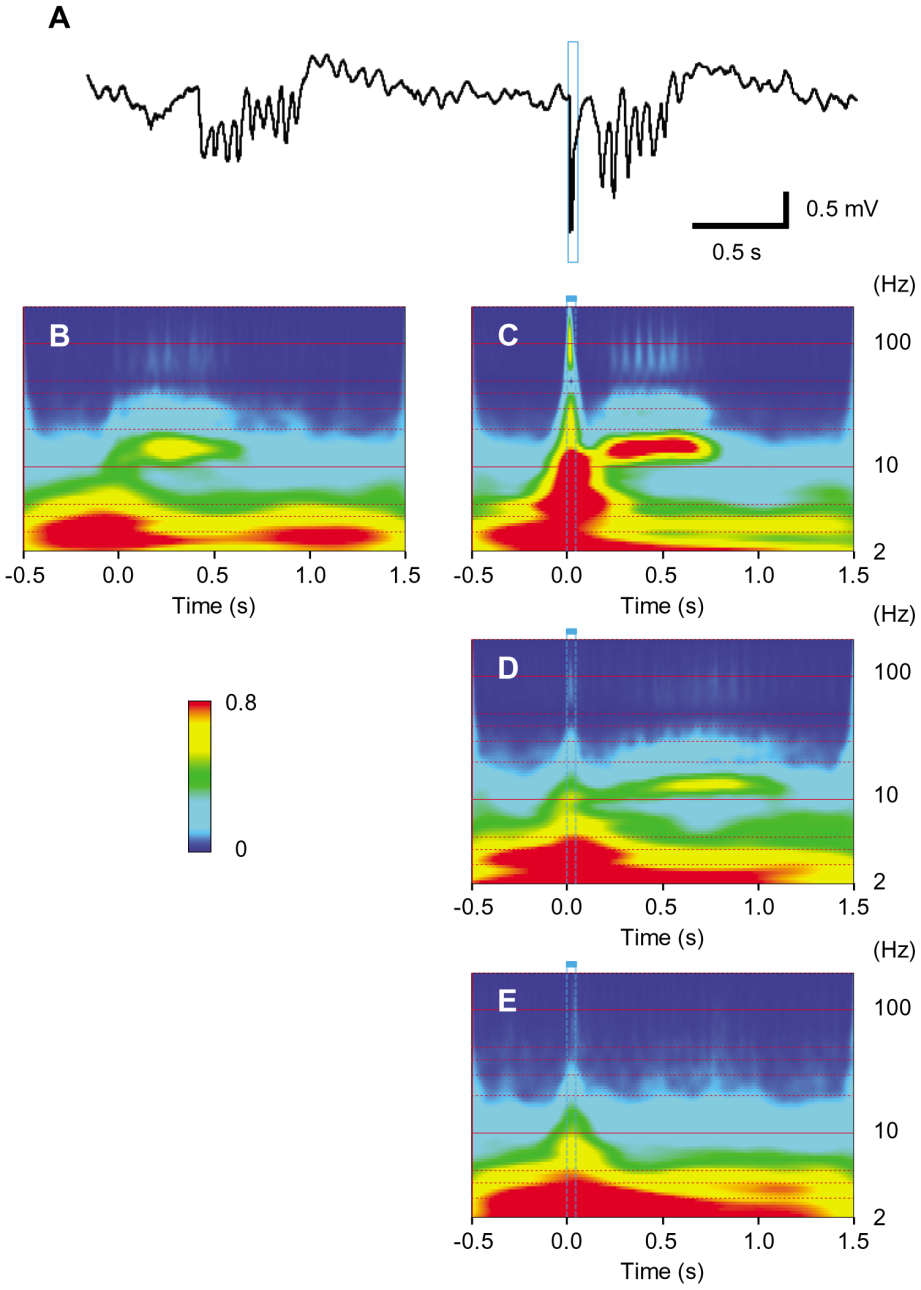
Cortical oscillations. **A**, The spontaneous LFP oscillation (left) and the light-evoked oscillation (right). **B**, Wavelet analysis of spontaneous oscillation (n = 30). The timing of each oscillation was adjusted to the first peak. **C-E**, Wavelet analysis of the light-evoked responses; whisker pad irradiation (C, n = 30), single D3 whisker irradiation (D, n = 30) and single D6 whisker irradiation (E, n = 30).

**Figure 8:**
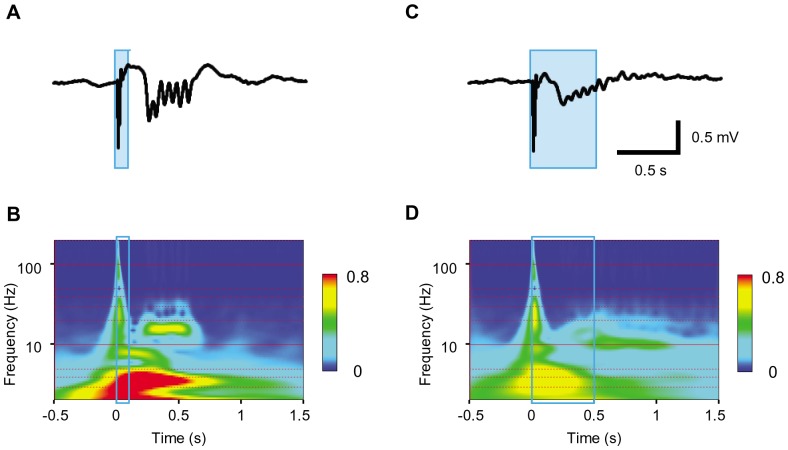
Cortical oscillations generated by long irradiation. **A**, The light-evoked LFP oscillation (duration, 100 ms). **B**, Wavelet analysis of the light-evoked responses during 100-ms whisker pad irradiation. C, The LFP response of the same recording site, but the irradiation was given for 500 ms. D, Wavelet analysis of above.

In the LFP recordings the oscillation at 10–20 Hz corresponded with the spontaneous bursts (Fig. 7A and 7B). Similar oscillations followed after the light-evoked LFP deflection by whisker-pad (Fig. 7C) or D3 irradiation (Fig. 7D) but not by D6 irradiation (Fig. 7E). The LFP oscillation at 10–20 Hz was robustly evoked in the other 7 experiments of whisker pad irradiation of a short duration (50–100 ms) and was typically as illustrated in Fig 8A. However, the power at 10–20 Hz deteriorated with the irradiation of a longer duration (500 ms) (Fig. 8B, n = 2) or with the mechanical stimulation by a short air puff (Figure S4, n = 5).

### Functional MRI study

**Figure 9:**
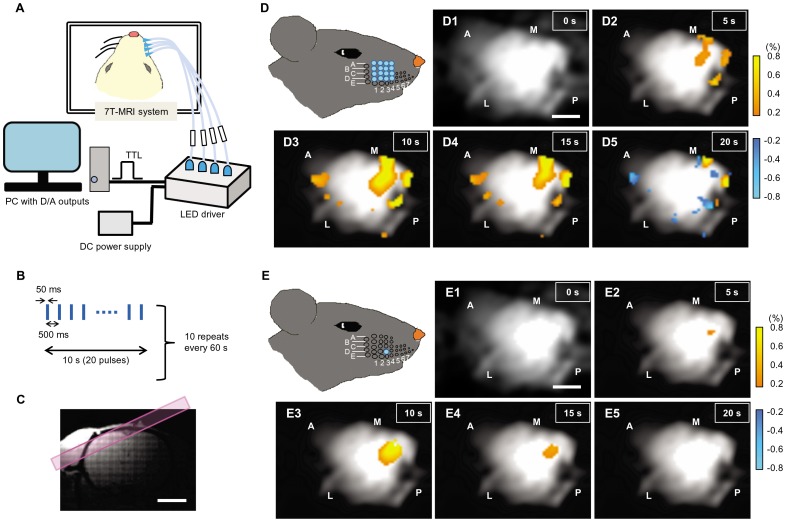
fMRI study of the optogenetic tactile sense (OTS). **A**, System setup. **B**, The block design of the stimulation paradigm. **C**, Coronal T1 image of the rat brain. The region selected for getting the tangential images is shown by a magenta stripe. **D**, Sequential images indicating the change of BOLD signal (*Δ*BOLD) induced by 16-whisker photostimulation. The time from onset of the first light pulse is indicated; 0 s (D1), 5 s (D2), 10 s (D3), 15 s (D4) and 20 s (D5). **E**, Sequential images indicating the*Δ*BOLD induced by a single D3 whisker photostimulation. The time from onset of the first light pulse is indicated; 0 s (E1), 5 s (E2), 10 s (E3), 15 s (E4) and 20 s (E5). Scale bars indicate 2 mm.

After trimming all whiskers and intervibrissal fur, an arrangement of 16 whisker follicles (A1-4, B1-4, C1-4, D1-4) of the right side was transcribed to a small acrylic plate. The acrylic plate was perforated in accordance with the arrangement of the 16 whisker follicles. On the day of the fMRI experiment, the rat was first anesthetized with isoflurane and then with intraperitoneal injection of urethane (1.3 g/kg body weight). The perforated acrylic plate was glued onto the whisker pad, adjusting each hole to each follicle, and connected to fiber-coupled LEDs in the 7T-MRI system (Fig. 9A). The fMRI measurement was performed with a block-designed stimulation paradigm consisting of 10 blocks. Each block was comprised of a 10 s whisker-photostimulation (50 ms at 2 Hz) followed by a 50 s resting condition (Fig. 9B) as the fidelity of the generating action potentials was expected to be high with this paradigm (Figure S5). Thus, the blue light pulses were given synchronously to the 16 whisker follicles at 10 mW/mm^2^ and the significant change of the BOLD signal (*Δ*BOLD) was tangentially imaged (Fig. 9C). Figure 9D1-5 shows the sequential images of a typical *Δ*BOLD. Positive *Δ*BOLD voxels emerged multiply in the contralateral barrel field as early as 5 s later (Fig. 9D2), spread rapidly in 10 s (Fig. 9D3) and disappeared with some negative *Δ*BOLD in regions outside the barrel field (Fig. 9D5). The *Δ*BOLD was also induced even by a single D3 whisker photostimulation in a more restricted region (Fig. 9D1-5). In summary (Table S1), the positive *Δ*BOLD (p<0.05) was induced in the contralateral cortex of 4/4 ChR2V+ rats by the 16-whisker photostimulation and of 3/4 ChR2V+ rats by the single D3 whisker photostimulation, but in the ipsilateral cortex of only 1/4 ChR2V+ rats. On the other hand, no significant *Δ*BOLD was evoked in the contralateral cortex of 4/4 non-transgenic (ChR2V-) rats.

## Discussion

### Expression of ChR2V in the mechanoreceptive neurons in TG

The TG neurons innervating whiskers project their axons to the spinal trigeminal nucleus, the second-order, so-called barrelettes in the caudal brain stem. Then the axons of second-order neurons cross the brain midline and travel to the thalamic somatosensory nuclei—the third-order neurons, so-called barreloids, and finally primary somatosensory cortex, mainly layer IV cells called “barrels” [1], [3]. In the W-ChR2V4 rat, the ChR2V+ TG neurons were mostly myelinated because of the co-expression of NF200. They were generally large in somal size (diameter > 24 μm). Although they negligibly co-expressed CGRP, a marker of peptidergic nociceptive neurons, the small occurrence of TG neurons expressing both ChR2V and P2X_3_ (7%) was relatively larger than that in DRG (4.3%). It is possible that these large P2X_3_-positive neurons are involved in noxious sense in the tooth pulp or muscle, but not in cutaneous nociception [8], [9].

### Expression of ChR2V in the mechanoreceptors of whisker follicle

In the whisker pad, ChR2V+ nerve fibers were mostly myelinated with the co-expression of NF200 or MBP (Figure S6). As their endings were associated with Merkel cells throughout the FSC, they were assumed to be involved in touch-pressure [7], [10]. All these findings suggest that the mechanoreceptive nerve endings around the whisker follicles expressed ChR2V.

### ChR2V+ TG neurons are responsive to light

The whole cell recording data showed that the ChR2-expressing TG neurons generated photocurrent or action potentials in response to blue light irradiation. Recently, on the basis of the evoked firing pattern, the adult rodent TG neurons have been assigned to one of three physiologically defined classes: the multiple-firing (MF) neurons, the delayed multiple-firing (DMF) neurons, and the single-spiking (SS) neurons [11]. The SS neurons are fast-adapting and fire only a single action potential regardless of the stimulating intensity. In the present results, all ChR2V+ neurons fired only one action potential, a trait frequently observed in the touch-sensitive neurons in the somatosensory system [12]. In the case of mechanoreception, the mechanical force evokes the deformation of the membrane of peripheral nerve endings to facilitate the opening of a certain type of channels [13], [14]. As a result, the membrane depolarization activates voltage-dependent ion channels, such as Na^+^ channels. In the present case, the light absorption depolarized the membrane directly through activating ChR2 channels, thus manipulating the activities of TG neurons. With the rapid rise of ChR2 photocurrent as well as with the membrane properties of TG neuron, the action potential was almost phase-locked to the onset of the LED pulse with millisecond-order precision.

Neurons in the TG consist of diverse populations that are involved in proprioception, touch, nociception and thermal sensation. This idea is supported by electrophysiological studies. Action potentials (APs) of different durations and amplitudes have been described previously [15], [16]. More recently, with whole-cell recording on acutely dissociated cells, Xu *et al* classified nine subtypes based on the current signature. Of them, five types of cells have larger cell bodies. And four larger type cells showed long-duration APs, while the remaining one had short-duration APs [17]. Such variable electrophysiological behavior is probably dependent on the different membrane properties generated by ion channels. For example, there are reports suggesting that I_A_ (transient A-type K^+^ current), I_D_ (dendrotoxin-sensitive K^+^ current), and I_M_ (M current by K_v_7.2-7.5) contributed to the APs in larger TG neurons [18]–[20]. The current formed by K^+^ channels is important for the AP duration and adaptation. Of course, the difference in Na^+^ and Ca^2+^ channel subtypes should also be crucial for the shapes and amplitudes of the AP [21]. Our cells expressing ChR2 are larger cells that would consist of several subpopulations involved in proprioception, touch or vibration. Since these cells possess diverse ion channels responsible for distinct functions, it is not surprising that the APs of ChR2-expressing neurons evoked by light showed different amplitudes, although the expression of ChR2 may alter the membrane properties of these neurons. As the photoactivated ChR2 pass cations rather non-selectively [23], it would increase the membrane conductance, thus reduce the action potential or block the invasion of action potential from the axon.

### Light-evoked whisker-barrel responses

Based on the 10% energy penetration through the skin, the perifollicular nerve endings are presumed to have been irradiated at 0.4–0.5 mWmm^−2^ in the present experiments. This power is estimated to generate ∼60% of the peak and ∼80% of the steady state of the maximal ChR2 photocurrent as their *K_m_* values were 0.26 and 0.085 mWmm^−2^ for the peak and the steady-state photocurrents, respectively (Fig. 3B). It would also be expected to generate action potentials in ∼70% of the TG neurons (Fig. 3E), although the excitability of the peripheral nerve endings *in vivo* should be different from that of cultured neurons.

Indeed, cortical responses such as MUA and LFP were detectable in the barrel field of ChR2V+ transgenic rat in response to blue light irradiation on the contralateral whisker pad, but not in response to red light or irradiation on the ipsilateral whisker pad. Therefore, these responses were suggested to be initiated by the depolarization of ChR2V+ nerve endings innervating the whisker follicles. This assumption was also evidenced by the fact that similar cortical responses were evoked by blue light irradiation confined to a single whisker follicle through a fiber-coupled LED. Since the ChR2V was selectively expressed in the large mechanoreceptive TG neurons, it is assumed that the rat sensed the spatiotemporally patterned blue light as a patterned touch to its whiskers. We would refer to this an “optogenetic tactile pattern (OTP)”.

### Cortical network responses to OTP

The OTP enables one to correlate the cortical responses to the whisker stimulation in the order of milliseconds [22], [23]. Indeed the cortical neurons responded to single whisker photostimulation with a short onset latency of 11.9±0.86 ms, a value that is consistent with those of previous studies using the mechanical deflection of a whisker [24], [25]. However, the latency was reduced to 6.8±0.60 ms in the case of whisker-pad irradiation, which would stimulate many principle and adjacent whiskers at once. The LEB also reached the maximum earlier by the whisker-pad irradiation than by the single whisker irradiation. It is possible that the synaptic inputs driven by the surrounding whiskers converged in the cortical neurons through the posterior medial (POm) nucleus of the thalamus to enhance the EPSP by the thalamocortical inputs from the ventral posterior medial (VPM) nuclei, thus accelerating the spike initiations [26]–[28]. Therefore, the surrounding whiskers appear to be involved in timing the detection of touch. The synchronous touch, such that by a large obstacle, would enhance the timing discrimination. On the other hand, the latency was prolonged in the case of mechanical stimulation by a short air puff (21±1.00 ms). This can possibly be attributed to the asynchronous deflection of whiskers, although the mechanical delay of pressure should be taken into consideration.

In the present study the whisker photostimulation was frequently associated with spindle-burst oscillatory activities of the cortical neurons. The bursts occurred rhythmically with 10–20 Hz (alpha-beta band) but did not continue over 1 s. Similar spindle-burst oscillatory activities also occurred spontaneously and are reminiscent of the spontaneous burst clusters prominent during non-REM sleep and anesthesia [29], [30]. This oscillatory activity appears to be dependent on the synchronous input of afferents as it is typically evoked by a short irradiation (50–100 ms). On the other hand, the cortical input may be asynchronous in the case of longer irradiation (500 ms) or mechanical stimulation by a short air puff. These oscillatory activities are based on the corticothalamic rhythms during the Up state of the cortical network [31]–[33]. It was reported previously that the barrel cortical response to whisker deflection was dependent on the slow oscillation and was more prominent during the Down state [34]–[36], although it was also dependent on the stimulus characteristics [37]. In the present study the LEB was robustly associated with quiescence and then followed by the spindle-shaped PAB, although the typical cycle of Up and Down states was not apparent. It is hypothesized that the photostimulation-dependent synchronous thalamocortical inputs evoked the initial bursting activity of the cortical neurons, which induces the transition of the cortical state from Up to Down [38]. The activation of cortical neurons also induces an intra-thalamic rhythm in parallel [32]. As the photostimulation-induced Down state opens the gate for the thalamocortical inputs [39], the spindle bursts occurs after quiescence.

The LEB peaked earlier by the whisker-pad irradiation than by the single-whisker irradiation. The duration of LEB was also dependent on the irradiation site. In addition, the photostimulation-evoked PAB was more robust, higher in magnitude and more prolonged in response to the whisker-pad irradiation than in response to the single-whisker irradiation. The PAB magnitude was variable from trial to trial, but showed no trends with repeated irradiation at 0.05 Hz (Figure S7A). On the other hand, the inter-burst interval was relatively stable (Figure S7B). The robustness, the magnitude and the duration of the spindle burst were even dependent on the irradiating site. Therefore, a different spatial pattern of irradiation evoked a different activity pattern in the cortical response.

To investigate the cortical response to OTP we measured in the present study the extracellular recordings such as MUA and LFP. For example, the high magnitude of MUA calculated from SD_MUA_ (Figure S3) indicates that many neurons near the electrode are synchronously firing. On the other hand the quiescent phase just after the LEB indicates that these neurons are synchronously subthreshold for generating action potentials. Therefore, we could not know about subthreshold (synaptic and/or membrane excitability) responses of a neuron by MUA. Although the LFP is dependent on the local electric current generated by the synaptic and/or membrane excitability of active neurons, it cannot allow to monitor silent neurons whose subthreshold properties can be affected by OTP. The subthreshold neural responses derived from these silent neurons should be investigated in future with higher spatiotemporal resolutions using intracellular recordings such that by patch clamp. The sources and sinks of cortical local network have to be also identified in future by the current-source density analysis using multiple electrodes.

### fMRI dynamics to OTP

When the TG nerves in the whisker pad were stimulated either mechanically or electrically, the *Δ*BOLD was induced in the barrel cortex in a manner dependent on the spatiotemporal pattern of stimulation [40]–[42]. The *Δ*BOLD has been assumed to have a relationship to the neural activity, although the underlying mechanism has not been fully resolved [43]–[46]. In the present study of fMRI, the light-evoked *Δ*BOLD was induced in the barrel field and was time-dependent. This is consistent with the notion that the spatiotemporally patterned OTP to the whisker follicles produced spatiotemporally patterned cortical activity in the rat brain. Therefore, the OTP is applicable to non-invasive imaging techniques such as fMRI to reveal the altered neuronal activity and networks across an entire structure or even the whole brain [47]–[49], although the spatiotemporal resolution of fMRI should be improved in the future.

## Conclusion

The cortical response to a single whisker deflection is actually directionally selective, that is, the deflection to a preferential direction evokes a larger response than others [50]. Moreover, active whisking is involved in somatosensory object localization [51]–. The OTP is un-physiological in the sense that it is dependent on neither the direction of deflection nor on active whisking. On the other hand, given its simplicity and lack of nociception, the OTP should facilitate studies on how the spatiotemporal pattern is represented in the animal.

Over 50 years ago, Hubel, Wiesel and their colleagues investigated how the spatiotemporally patterned assembly of light-induced signals in the retina could be interpreted in the cerebral cortex as information about the shape, size, movement, color and depth of objects [54], [55]. In their series of experiments they measured the cortical responses while producing many sizes and shapes of spots of light and focused them on the animal's retina. Similarly, the spatiotemporal pattern of touching points is interpreted in the somatosensory cortex as information about the shape, size, weight, movement and texture of the touching objects. There remain many enigmas about these complex perceptions [3]. Where are they generated? How are they generated in the network? Are they generated by experience? The present paper should open a new avenue for investigating these unresolved questions by applying Hubel and Wiesel's strategy to the touch sense with the combination of OTP, electrophysiological and imaging techniques.

## Materials and Methods

### Animals

The experiments were carried out using offspring of one of the thy1.2 promotor-ChR2-*Venus* transgenic rat lines, W-TChR2V4 with the genetic background of Wistar rats [4]. All animal experiments were approved by the Tohoku University Committee for Animal Experiments (Approval No. 2013LsA-016) and were carried out in accordance with the Guidelines for Animal Experiments and Related Activities of Tohoku University as well as the guiding principles of the Physiological Society of Japan and the National institutes of health (NIH), USA. The number of animals in this study was kept to a minimum and, when possible, all animals were anesthetized to minimize their suffering. Animals had access to food and water ad libitum and were kept under a 12-hour light-dark cycle.

### Immunohistochemistry

ChR2-expressing W-TChR2V4 (ChR2V+) rats (five weeks old) were used for the immunohistochemical experiments as described previously [5]. Briefly they were anesthetized with a mixture (1 ml/kgBW) of ketamine (50 mg/ml, Daiichi Sankyo Co. Ltd., Tokyo, Japan) and xylazine (xylazine hydrochloride, 10 mg/ml, Sigma-Aldrich, St. Louis, MO, USA) and transcardially perfused with phosphate-buffered saline (PBS; pH 7.4), followed by 100 ml of 4% paraformaldehyde (PFA) and 0.2% picric acid in PBS. The TGs and whisker pad were removed and post-fixed in 4% PFA overnight at 4°C. After cryoprotection through a graded series of sucrose replacements (10, 20 and 30% in PBS) at 4°C, each tissue was embedded in OCT Compound (Sakura Finetek, Tokyo, Japan) and stored at −80°C.

The localization and cell type of ChR2V+ neurons in the tissue were immunohistochemically investigated using anti-EGFP antibody [56] along with the antibody of one of the cell-type-specific markers. Briefly, each frozen section was cut at 16 μm thickness with a cryostat (CM 3050S, Leica, Wetzlar, Germany), mounted on poly-L-lysine coated slides (Matsunami Glass Ind. Ltd., Kishiwada, Japan) and left to air-dry for 90 min at room temperature. After washing with PBS, slices were incubated for 1 hr in blocking PBS containing 2.5% goat serum, 0.25% carrageenan and 0.1% Triton X-100 at room temperature. Then, the specimens were reacted overnight at 4°C with the primary antibody: rabbit anti-EGFP (1∶2,000); mouse monoclonal anti-NF200 (1∶500, N0142, Sigma-Aldrich); rabbit anti-CGRP (1∶2,000, C8198, Sigma-Aldrich); guinea- pig anti-CGRP (1∶1000, Progen Biotechnik GmbH, Heidelberg, Germany); guinea- pig anti-P2X_3_ (1∶1,000, GP10108, Neuromics, Edina, MN, USA); chicken anti-MBP (1∶500, PA1-10008, Thermo Fisher Scientific K.K., Yokohama, Japan), and mouse anti-CK20 (1∶20, IT-Ks 20.8, Progen Biotechnik GmbH). In some specimens, the Venus fluorescence signal could be directly examined without any amplification. After 10-min washing three times, the slices were reacted for 1 hr (room temperature) or overnight (4°C) with a combination of the following secondary antibodies (Molecular Probes products from Life Technologies Co., Carlsbad, CA, USA, except for Dylight-549): Alexa Fluor 488-conjugated donkey anti-rabbit IgG (1∶500), Alexa Fluor 546-conjugated donkey anti-mouse IgG (1∶500), Alexa Fluor 546-conjugated donkey anti-rabbit IgG (1∶500) and Alexa Fluor 633-conjugated goat anti-guinea pig IgG (1∶500), and Dylight 549-conjugated goat anti-chicken IgY (1∶100; Thermo Fisher Scientific K.K., Waltham, MA, USA). After washing three times in PBS, the specimens were mounted with PermaFluor (Thermo Fisher Scientific K.K.). Images were digitally captured under conventional confocal laser-scanning microscopy (LSM510META, Carl Zeiss, Oberkochen, Germany) and were corrected for brightness and contrast using LSM Image Browser version 3.2 (Carl Zeiss), Photoshop version 6.0 (Adobe Systems Inc, San Jose, CA, USA) and ImageJ (http://rsbweb.nih.gov/ij/). The diameter of a TG neuron was microscopically measured as the mean of the shortest and longest diameters.

### Primary culture and patch clamp

The TG neurons of ChR2V+ rats (3–4 weeks old) were cultured according to a method reported previously [5] with some modifications. After decapitation, the two TGs were taken out and put into an ice-cold dissecting solution containing DMEM (D5030, Sigma-Aldrich) supplemented with 3.7 mg/ml NaHCO_3_, 1 mg/ml D-glucose, 2 mM L-glutamine (G7513, Sigma-Aldrich), 1% penicillin/streptomycin (P0781, Sigma-Aldrich). Each TG was cleaned of the surrounding connective tissue, cut into small pieces and immersed in an enzymatic solution containing 1.0 mg/ml collagenase II (C6885, Sigma-Aldrich), 0.5 mg/ml trypsin (15090-046, Life Technologies Co., Carlsbad, CA, USA) and 0.1 mg/ml DNase I (Sigma-Aldrich) for 30–45 min at 37°C. The cells were washed twice with trituration solution containing 2 mg/ml BSA (A7906, Sigma-Aldrich), re-suspended in culture medium containing DMEM (D5030, Sigma-Aldrich) supplemented with 3.7 mg/ml NaHCO_3_, 1 mg/ml D-glucose, 2 mM L-glutamine (G7513, Sigma-Aldrich), 1% penicillin/streptomycin (P0781, Sigma-Aldrich) and 10% FBS (04-001-1, Biological Industries, Beit-Haemek, Israel), and then plated and cultured at 37°C in a humidified incubator with a 95% air and 5% CO_2_ atmosphere. The culture medium was changed every two days. The whole-cell recording experiments were carried out within 4–5 days of plating.

The ChR2V+ TG neurons were identified under conventional epi-fluorescence microscopy (BH2-RFC, Olympus Optical Co., Tokyo, Japan) equipped with a 40× water-immersion objective (LUMplanP1/IR40x, Olympus) and a conventional filter cube (excitation, 495 nm; dichroic mirror, 505 nm; barrier filter, 515 nm). The electrophysiological recording was performed at 34±2°C (UTC-1000, Ampere Inc., Tokyo, Japan) under whole-cell patch clamp from the soma using an amplifier (EPC 8, HEKA Elektronik Dr. Schulze GmbH, Germany) and computer software (pCLAMP 9, Molecular Devices, LLC, Sunnyvale, CA). The bath solution was composed of (in mM) 138 NaCl, 3 KCl, 2 CaCl_2_, 1 MgCl_2_, 4 NaOH, 10 HEPES, 11 glucose, and was adjusted at pH 7.4 by 1 N HCl. The patch pipette solution was composed of (in mM) 125 K-gluconate, 10 KCl, 0.2 EGTA, 10 HEPES, 1 MgCl_2_, 3 MgATP, 0.3 Na_2_GTP, 10 Na_2_-phosphocreatine and 0.1 leupeptin, and was adjusted to pH 7.2 by 1 N KOH. The liquid junctional potential was directly measured as -11.5 mV and was compensated for. When the resting potential was > -55 mV or the input resistance was < 500 MΩ, the data were excluded from the following studies. For the optical actuation of a TG neuron we used a blue LED (470 ±25 nm wavelength, LXHL-NB98, Philips Lumileds Lighting Co., San Jose, CA, USA) regulated by a pulse generator (SEN-7203, Nihon Kohden, Tokyo, Japan) and computer software (pCLAMP 9, Molecular Devices, LLC). The maximal irradiance of the LED light was 1.6 mWmm^-2^ at the focus. The rise time of the photocurrent or action potential was measured from the onset of the LED pulse to the minimum or maximum of the first-order differentiation to time, respectively.

### *In vivo* whisker photostimulation and electrophysiological recording

Adult ChR2V+ rats (210–390 g) were used in this experiment. Surgical anesthesia was induced with isoflurane and maintained with intraperitoneal injection of urethane (1.3 g/kg body weight). All pressure points and incised tissues were injected with lidocaine. The anesthetic depth was monitored by observation of the hind-limb and eye reflexes. All whiskers were trimmed of the intervibrissal fur to photostimulate the trigeminal nerve endings innervating their follicles. Animals were placed on the stereotaxic apparatus with ear and tooth bars (David Kopf Instruments, Tujunga, CA, USA). For electrophysiological recordings, the skull was exposed and a small craniotomy was performed over the barrel cortex of left hemisphere (5.5 mm lateral, 2.5 mm rostral from the bregma). During surgical and recording procedures, the body temperature was maintained at 37°C using a homeothermic heating pad.

Multi-unit activity (MUA) and local field potential (LFP) were recorded in the barrel cortex using a tungsten microelectrode (0.8–1.4 MΩ at 1 kHz; FHC). The electrode was inserted perpendicularly into the cortex with a micromanipulator. MUA and LFP recordings were performed at a depth of 1 mm from the pial surface. Recorded signals were amplified with a biophysical amplifier (AVB-11A; Nihon Kohden) and bandpass filtered for MUA (300–3000 Hz) or LFP (0.5–300 Hz) and stored in the computer via an analog-digital converter (Digidata; Molecular Devices) at a sampling rate of 20 kHz.

A blue LED (470 nm, LXML-PB01-0023; Philips Lumileds Lighting Co. San Jose, CA, USA) and a red LED (625 nm, LR W5AP-LXMY-1; OSRAM Opto Semiconductors GmbH, Regensburg, Germany) were used for the whisker pad irradiation (50 ms light pulse). The distance between the LED and the skin was 0.5–1 cm with a light power density of 4–5 mWmm^−2^ (at the mystacial pad) for each LED light. For a single whisker follicle, the blue LED light (50 ms pulse) was irradiated through a plastic optical fiber (φ,750 μm, Edmund Optics Inc., Barrington, NJ, USA) with a light power density of 13 mWmm^−2^ (at the follicle surface). The light pulse irradiation was repeated 30 times at 0.05 Hz to obtain average MUA and LFP responses in all experiments.

### Analysis of electrophysiological data

All data were analyzed using Clampfit 10.2 (Molecular Devices) and Offline sorter (Plexon Inc., Dallas, TX, USA) software. All response data of each animal are the average of 30 consecutive trials of whisker irradiation. The MUA response is represented as peristimulus-time histogram (PSTH) with 1 ms bin. Time-dependent changes of the standard deviation of MUA (SD_MUA_) were calculated for each 5 ms-bin period and adopted as the MUA magnitude after subtraction of three times of the minimum SD_MUA_ (SD_min_) (Figure S3). Population data are expressed as mean ± SEM. Paired *t* -test was used for statistics unless otherwise noted.

### Functional magnetic resonance imaging (fMRI)

All MRI data were acquired from either non-transgenic (ChR2V-) or ChR2V+ rats using a 7.0-T Bruker PharmaScan system (Bruker Biospin Co., Ettlingen, Germany) as described previously [46]. A 72-mm birdcage transmit-only RF coil with an actively decoupled receive-only quadrature planar surface coil (10-mm diameter), which was placed just above the barrel cortex, was used. Prior to all of the MRI experiments, global magnetic field shimming was performed first inside the core and, later, completed at the region-of-interest (ROI) using a point-resolved spectroscopic protocol. The line width (full width at half maximum) at the end of the shimming procedure ranged from 15 to 20 Hz in the ROI (∼300 μl). The fMRI signal was obtained from the single tangential imaging plane parallel to the cortical surface using GE-EPI with the following parameters: TR = 1000 ms, TE = 15 ms, FOV = 30×30 mm^2^, matrix size  = 100×100, in-plane resolution  = 300×300 μm^2^, slice thickness  = 2 mm, and number of volumes  =  660. The fMRI signal was also obtained from the coronal imaging plane using GE-EPI with the following parameters: TR = 1000 ms, TE = 15 ms, FOV = 25.6×14.0 mm^2^, matrix size = 64×35, in-plane resolution = 400×400 μm^2^, slice thickness = 400 μm, number of slices = 20, and number of volumes = 660. The body temperature was monitored using an MRI-compatible temperature probe (SA Instruments, Inc., Stony Brook, NY, USA) inserted into the rectum. The core body temperature was carefully maintained at 37.0±1°C during the entire fMRI experiment by means of a heated water-circulating pad.

One end of a plastic optical fiber (φ, 750 μm, CK30, Mitsubishi Rayon Co. Ltd., Tokyo, Japan) was connected to the LED light source (FCS-0470-000, Mightex Systems, Toronto, Canada), while the other end was inserted into the hole with optical adhesive (SCR-1016A, Shin-Etsu Chemical Co., Ltd., Tokyo, Japan). The light power density at the other end of each fiber was adjusted to 10 mWmm^−2^ at the follicle surface. The light pulse was regulated with a 16-channel LED Controller (SLC-CA 16-, Mightex Systems).

The pre-processing of the fMRI data analysis was carried out using SPM8 (Wellcome Trust Centre for Neuroimaging, University of College London, London, UK) and custom-written software in MATLAB (The MathWorks Inc., Natick, MA, USA). The pre-processing of the fMRI data included correcting for head movement using the first EPI image and smoothing using a Gaussian kernel of 0.8 mm full width at half maximum. The single-subject analysis of the preprocessed fMRI data was performed using SPM8 with a critical T-value for each voxel (*p*<0.001, *p*<0.01, and *p*<0.001, uncorrected for multiple comparisons). The resulting T-map of the fMRI data was overlaid on the mean EPI image of each subject.

## Supporting Information

Figure S1 Relationship between the threshold irradiance and the input conductance. Each point represents a TG neuron for which the minimal irradiance necessary to generate an action potential (threshold irradiance) was related to its input conductance, a reciprocal of input resistance with a correlation coefficient of 0.77 (n = 16). The broken line, *y* = 0.70 *x* -0.16, is the least-squares fitting to the linear relationship.
(PDF)

Figure S2 Early generation of action potentials. The same typical data as shown in Fig. 3D, but on an expanded time scale. Note that the action potential was evoked within 10 ms irradiation even at the threshold strength.
(PDF)

Figure S3 SD_MUA_ analysis. **A**, The MUA data shown in text-Fig. 4A was re-plotted as SD_MUA_. **B**, Each SD was calculated for a duration of 5 ms (100 consequent points). (1) The MUA magnitude was measured over time as the SD_MUA_ minus 3 times of the minimal SD_MUA_ (SD_min_) during 1 s-period after the light onset. (2) Time to peak LEB: the time to reach the peak SD_MUA_ of LEB from the onset of blue irradiation. (3) LEB duration: the duration above 3 times of the SD_min_. (4) PAB duration: the duration above 3 times of the SD_min_ during the afterburst. (5) Interburst interval: the interval between a peak SD_MUA_ and the next.
(PDF)

Figure S4 Cortical response to the mechanical stimulation of whiskers. **A**, A typical MUA response to 50-ms air puff stimulation (red bar) of the contralateral whiskers. Note the spontaneous bursts and after-stimulus bursts. **B**, The LFP data of the same period as in A. **C**, Wavelet analysis of the air-puff-evoked responses (n = 30).
(PDF)

Figure S5 Fidelity of TG neuron firing to repetitive photostimulation. **A**, When short LED pulses (duration, 20 ms) were repeatedly applied, the first pulse robustly evoked action potentials at the maximal irradiance (1.6 mWmm^−2^). However, the following LED pulses occasionally failed to evoke action potentials and the average fidelity was reduced with the increase of frequency. **B**, Fidelity of generation of action potentials as a function of frequency (mean ± SEM, n = 14). The number of neurons that have no failure during repetitive stimulation was 13/14 at 1 Hz, 7/14 at 2 Hz, 4/14 at 5 Hz, 4/14 at 10 Hz and 1/14 at 20 Hz.
(PDF)

Figure S6 ChR2+ nerve endings innervating whisker follicle. **A**, Immunohistochemical identification of the ChR2V+ nerve endings surrounding the whisker follicle (middle layer) with markers for myelinated axons: NF200. **B**, Similary the co-expression of ChR2V and myeline basic protein (MBP, B). ICB, inner conical body; ORS, outer root sheath. Scale bars, 20 μm.
(PDF)

Figure S7 Effects of repeated photostimulation on the PAB dynamics. **A**, The PAB magnitude was plotted sequentially with photostimulation by blue LED (50 ms, 0.05 Hz) on the contralateral whisker pad (dark blue) or on the D3 whisker follicle (light blue). **B**, The average inter-burst interval was plotted sequentially in the same experiment as in A.
(PDF)

Table S1 Summary of the functional MRI (fMRI) experiments.
(PDF)
