# Supplementary material for: Optogenetic Patterning of Whisker-Barrel Cortical System in Transgenic Rat Expressing Channelrhodopsin-2
Source: PLoS One. 2014 Apr 2;9(4):e93706. doi: 10.1371/journal.pone.0093706 (PMC3973546; doi:10.1371/journal.pone.0093706)
Supplement: Figure S1 — Relationship between the threshold irradiance and the input conductance. Each point represents a TG neuron for which the minimal irradiance necessary to generate an action potential (threshold irradiance) was related to its input conductance, a reciprocal of input resistance with a correlation coefficient of 0.77 (n = 16). The broken line, y = 0.70 x -0.16, is the least-squares fitting to the linear relationship. (PDF) [file pone.0093706.s001.pdf]

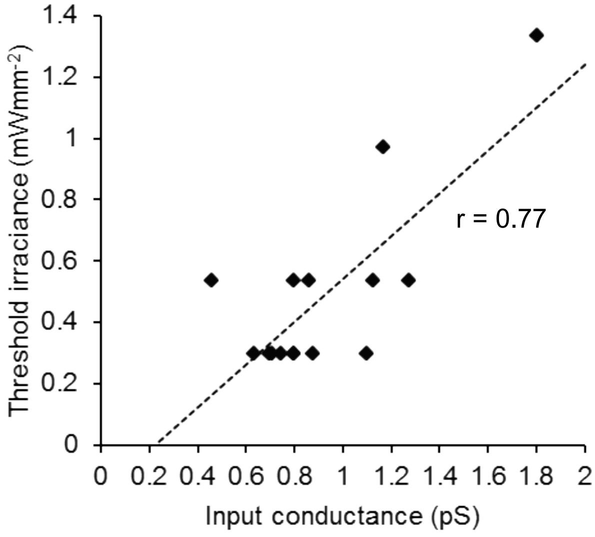

Figure S1. Relationship between the threshold irradiance and the input conductance.

Each point represents a TG neuron for which the minimal irradiance necessary to generate an action potential (threshold irradiance) was related to its input conductance, a reciprocal of input resistance with a correlation coefficient of 0.77 (n = 16). The broken line, y = 0.70 x -0.16, is the least-squares fitting to the linear relationship.
