# Supplementary material for: Optogenetic Patterning of Whisker-Barrel Cortical System in Transgenic Rat Expressing Channelrhodopsin-2
Source: PLoS One. 2014 Apr 2;9(4):e93706. doi: 10.1371/journal.pone.0093706 (PMC3973546; doi:10.1371/journal.pone.0093706)
Supplement: Figure S2 — Early generation of action potentials. The same typical data as shown in Fig. 3D, but on an expanded time scale. Note that the action potential was evoked within 10 ms irradiation even at the threshold strength. (PDF) [file pone.0093706.s002.pdf]

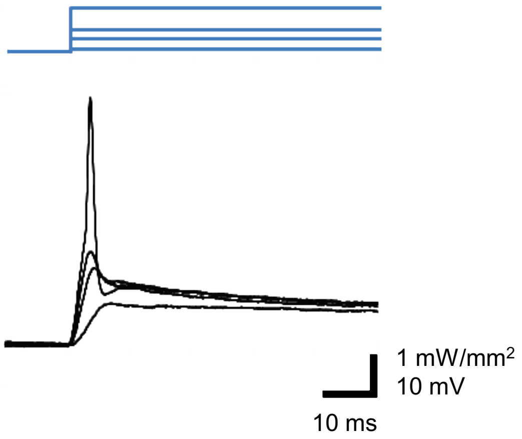


**Figure S2. Early generation of action potentials.**

The same typical data as shown in Fig. 3D, but on an expanded time scale. Note that the action potential was evoked within 10 ms irradiation even at the threshold strength.
