# Supplementary material for: Optogenetic Patterning of Whisker-Barrel Cortical System in Transgenic Rat Expressing Channelrhodopsin-2
Source: PLoS One. 2014 Apr 2;9(4):e93706. doi: 10.1371/journal.pone.0093706 (PMC3973546; doi:10.1371/journal.pone.0093706)
Supplement: Figure S3 — SDMUA analysis. A, The MUA data shown in text-Fig. 4A was re-plotted as SDMUA. B, Each SD was calculated for a duration of 5 ms (100 consequent points). (1) The MUA magnitude was measured over time as the SDMUA minus 3 times of the minimal SDMUA (SDmin) during 1 s-period after the light onset. (2) Time to peak LEB: the time to reach the peak SDMUA of LEB from the onset of blue irradiation. (3) LEB duration: the duration above 3 times of the SDmin. (4) PAB duration: the duration above 3 times of the SDmin during the afterburst. (5) Interburst interval: the interval between a peak SDMUA and the next. (PDF) [file pone.0093706.s003.pdf]

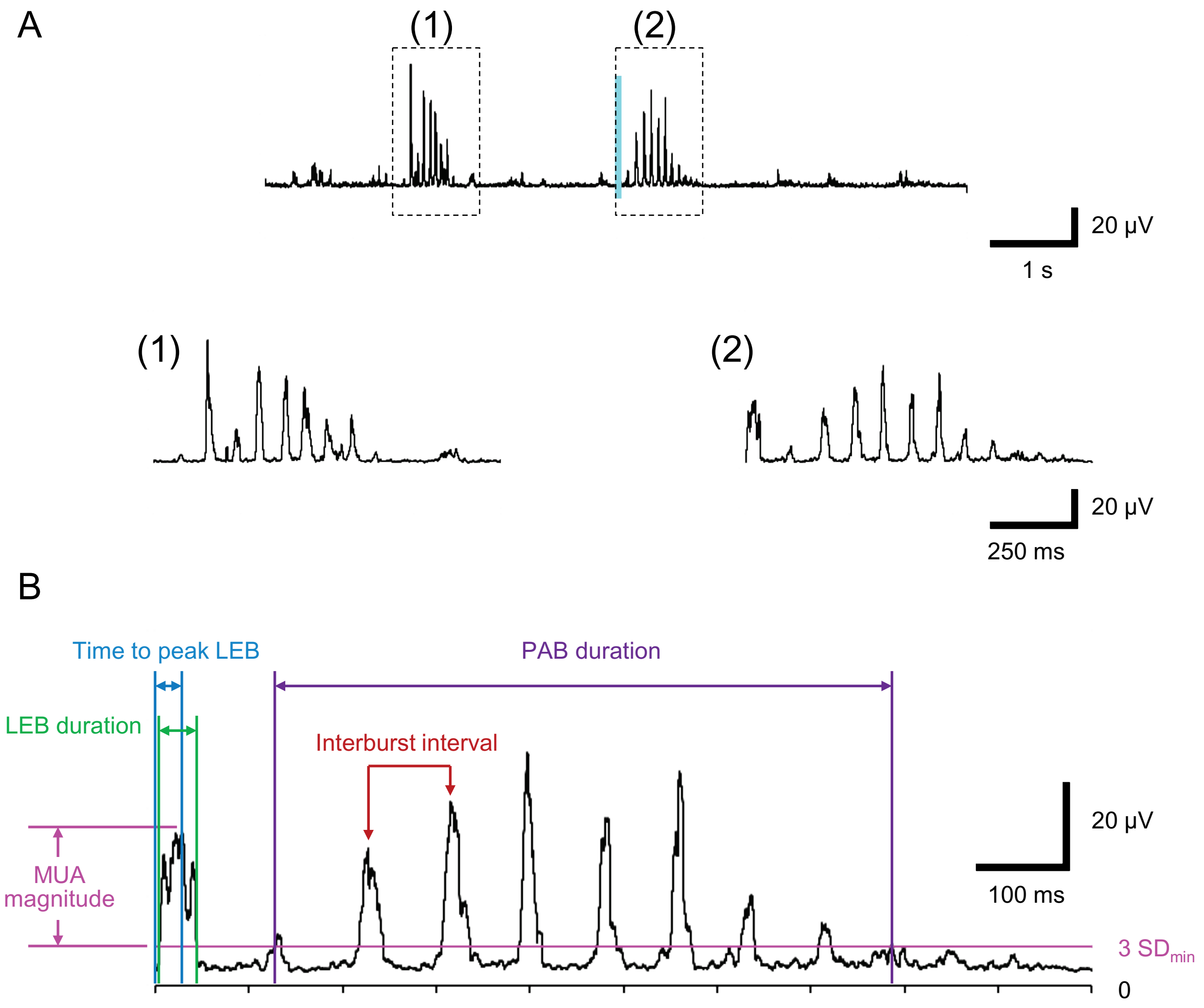

Figure S3. SD_{MUA} analysis.

A, The MUA data shown in text-Fig. 4A was re-plotted as SD_{MUA}. **B,** Each SD was calculated for a duration of 5 ms (100 consequent points).

(1) The MUA magnitude was measured over time as the SD_{MUA} minus 3 times of the minimal SD_{MUA} (SD_{min}) during 1 s-period after the light onset.

(2) Time to peak LEB: the time to reach the peak SD_{MUA} of LEB from the onset of blue irradiation.

(3) LEB duration: the duration above 3 times of the SD_{min}.

(4) PAB duration: the duration above 3 times of the SD_{min} during the afterburst.

(5) Interburst interval: the interval between a peak SD_{MUA} and the next.
