# Supplementary material for: Optogenetic Patterning of Whisker-Barrel Cortical System in Transgenic Rat Expressing Channelrhodopsin-2
Source: PLoS One. 2014 Apr 2;9(4):e93706. doi: 10.1371/journal.pone.0093706 (PMC3973546; doi:10.1371/journal.pone.0093706)
Supplement: Figure S4 — Cortical response to the mechanical stimulation of whiskers. A, A typical MUA response to 50-ms air puff stimulation (red bar) of the contralateral whiskers. Note the spontaneous bursts and after-stimulus bursts. B, The LFP data of the same period as in A. C, Wavelet analysis of the air-puff-evoked responses (n = 30). (PDF) [file pone.0093706.s004.pdf]

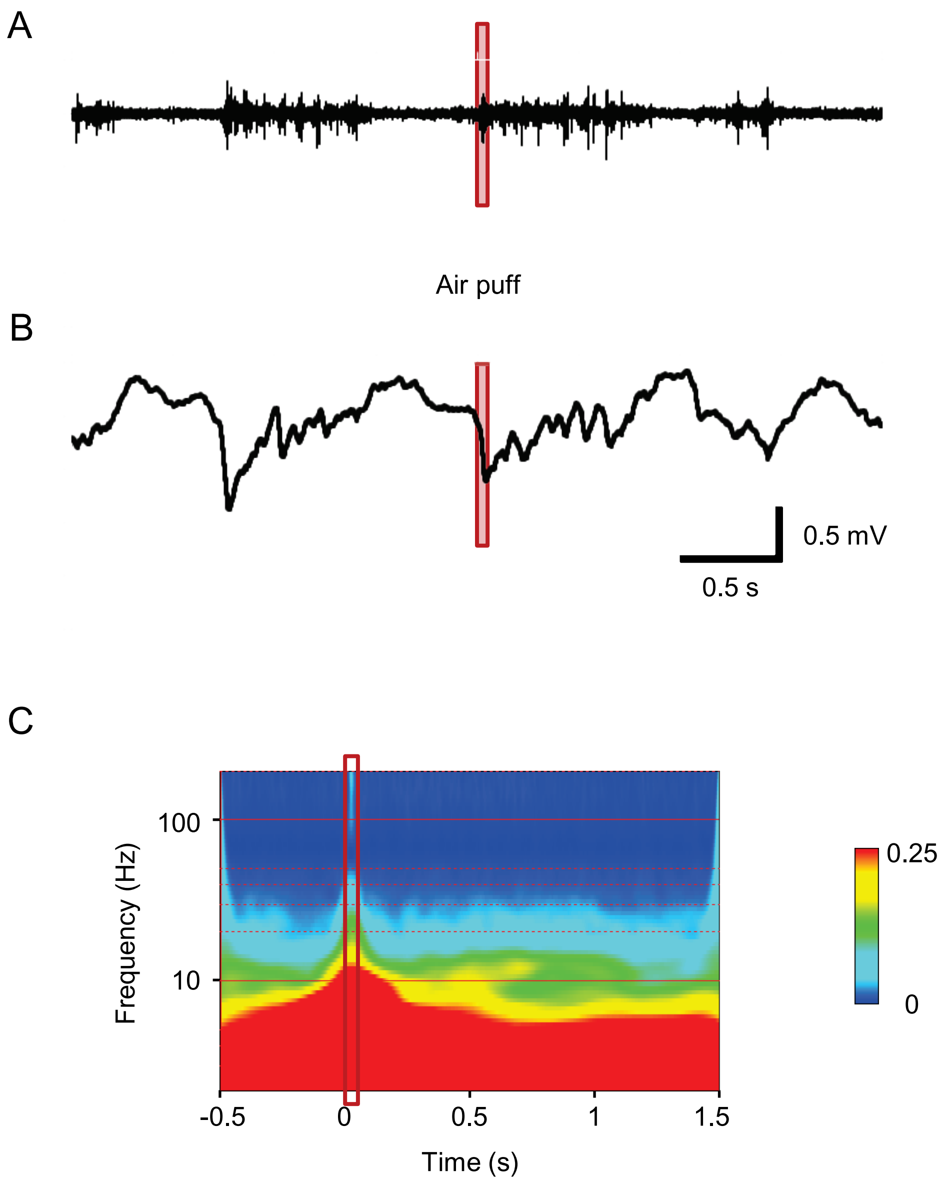

Figure S4. Cortical response to the mechanical stimulation of whiskers. **A,** A typical MUA response to 50-ms air puff stimulation (red bar) of the contralateral whiskers. Note the spontaneous bursts and after-stimulus bursts. **B,** The LFP data of the same period as in A. **C,** Wavelet analysis of the air-puff-evoked responses (n = 30).
