# Supplementary material for: Optogenetic Patterning of Whisker-Barrel Cortical System in Transgenic Rat Expressing Channelrhodopsin-2
Source: PLoS One. 2014 Apr 2;9(4):e93706. doi: 10.1371/journal.pone.0093706 (PMC3973546; doi:10.1371/journal.pone.0093706)
Supplement: Figure S5 — Fidelity of TG neuron firing to repetitive photostimulation. A, When short LED pulses (duration, 20 ms) were repeatedly applied, the first pulse robustly evoked action potentials at the maximal irradiance (1.6 mWmm−2). However, the following LED pulses occasionally failed to evoke action potentials and the average fidelity was reduced with the increase of frequency. B, Fidelity of generation of action potentials as a function of frequency (mean ± SEM, n = 14). The number of neurons that have no failure during repetitive stimulation was 13/14 at 1 Hz, 7/14 at 2 Hz, 4/14 at 5 Hz, 4/14 at 10 Hz and 1/14 at 20 Hz. (PDF) [file pone.0093706.s005.pdf]

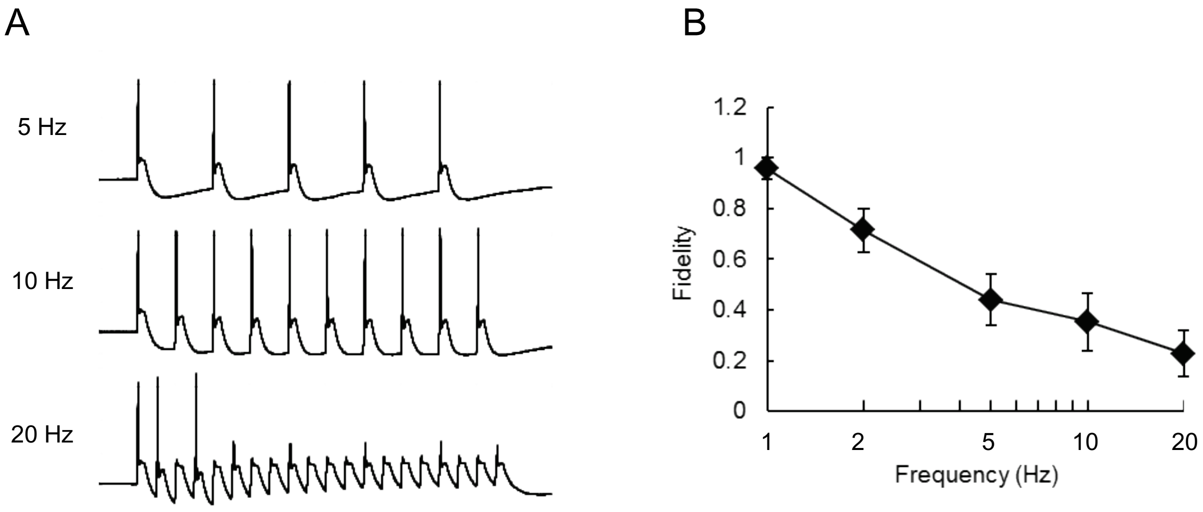

Figure S5. Fidelity of TG neuron firing to repetitive photostimulation.

A, When short LED pulses (duration, 20 ms) were repeatedly applied, the first pulse robustly evoked action potentials at the maximal irradiance (1.6 $mWmm^{-2}$). However, the following LED pulses occasionally failed to evoke action potentials and the average fidelity was reduced with the increase of frequency . **B,** Fidelity of generation of action potentials as a function of frequency (mean $\pm$ SEM, n = 14). The number of neurons that have no failure during repetitive stimulation was 13/14 at 1 Hz, 7/14 at 2 Hz, 4/14 at 5 Hz, 4/14 at 10 Hz and 1/14 at 20 Hz.
