# Supplementary material for: Optogenetic Patterning of Whisker-Barrel Cortical System in Transgenic Rat Expressing Channelrhodopsin-2
Source: PLoS One. 2014 Apr 2;9(4):e93706. doi: 10.1371/journal.pone.0093706 (PMC3973546; doi:10.1371/journal.pone.0093706)
Supplement: Figure S6 — ChR2+ nerve endings innervating whisker follicle. A, Immunohistochemical identification of the ChR2V+ nerve endings surrounding the whisker follicle (middle layer) with markers for myelinated axons: NF200. B, Similary the co-expression of ChR2V and myeline basic protein (MBP, B). ICB, inner conical body; ORS, outer root sheath. Scale bars, 20 μm. (PDF) [file pone.0093706.s006.pdf]

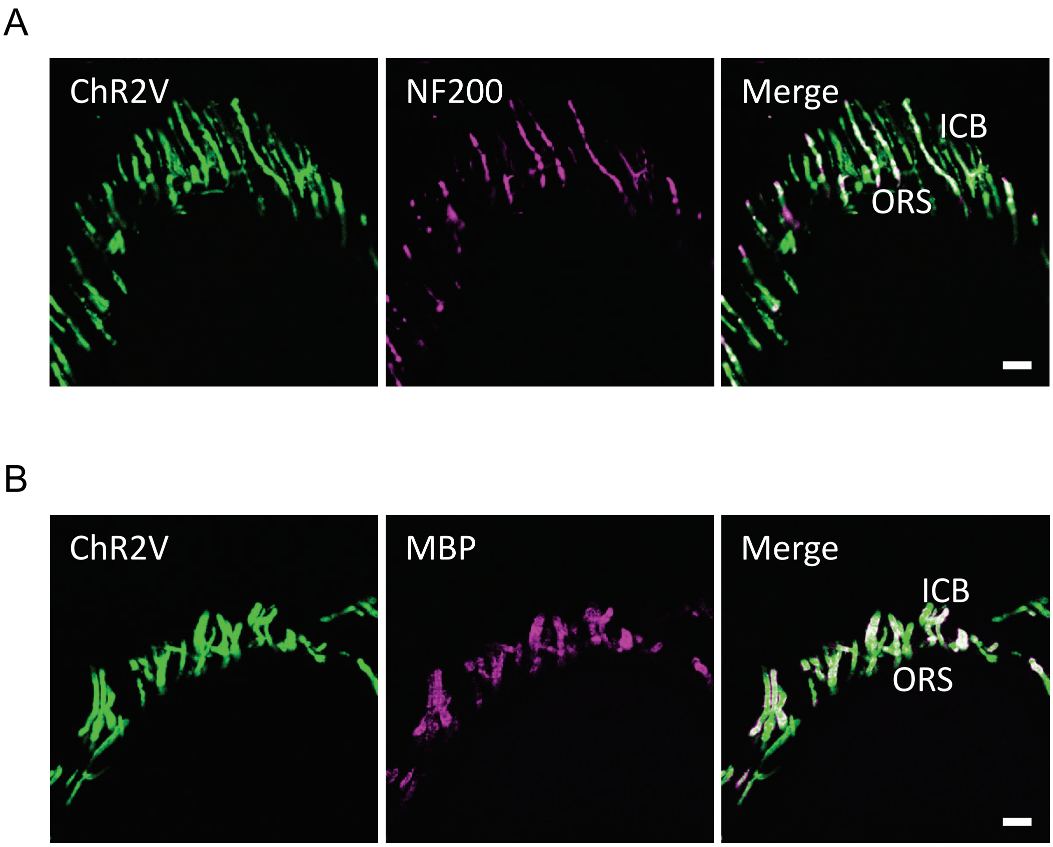

Figure S6. ChR2+ nerve endings innervating whisker follicle. A, Immunohistochemical identification of the ChR2V+ nerve endings surrounding the whisker follicle (middle layer) with markers for myelinated axons: NF200. **B,** Similary the co-expression of ChR2V and myeline basic protein (MBP, B). ICB, inner conical body; ORS, outer root sheath. Scale bars, 20 µm.
