# Supplementary material for: Optogenetic Patterning of Whisker-Barrel Cortical System in Transgenic Rat Expressing Channelrhodopsin-2
Source: PLoS One. 2014 Apr 2;9(4):e93706. doi: 10.1371/journal.pone.0093706 (PMC3973546; doi:10.1371/journal.pone.0093706)
Supplement: Figure S7 — Effects of repeated photostimulation on the PAB dynamics. A, The PAB magnitude was plotted sequentially with photostimulation by blue LED (50 ms, 0.05 Hz) on the contralateral whisker pad (dark blue) or on the D3 whisker follicle (light blue). B, The average inter-burst interval was plotted sequentially in the same experiment as in A. (PDF) [file pone.0093706.s007.pdf]

A

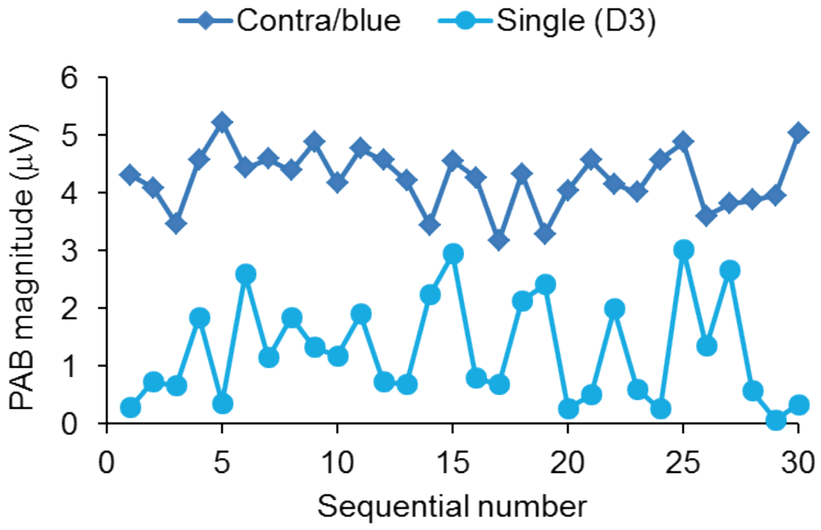

B

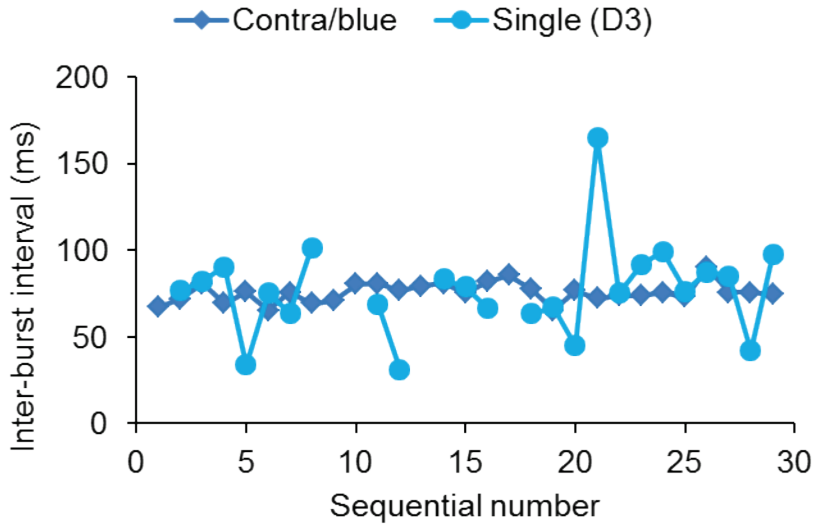

Figure S7. Effects of repeated photostimulation on the PAB dynamics.

A, The PAB magnitude was plotted sequentially with photostimulation by blue LED (50 ms, 0.05Hz) on the contralateral whisker pad (dark blue) or on the D3 whisker follicle (light blue). **B,** The average inter-burst interval was plotted sequentially in the same experiment as in A.
