# Supplementary material for: Optogenetic Patterning of Whisker-Barrel Cortical System in Transgenic Rat Expressing Channelrhodopsin-2
Source: PLoS One. 2014 Apr 2;9(4):e93706. doi: 10.1371/journal.pone.0093706 (PMC3973546; doi:10.1371/journal.pone.0093706)
Supplement: Table S1 — Summary of the functional MRI (fMRI) experiments. (PDF) [file pone.0093706.s008.pdf]

Table S1. Summary of the functional MRI (fMRI) experiments.

Animal	Side	Stimulation pattern	Scanning mode	Number of examined rats	Number of rat with a significant ⊿BOLD		
					$p<0.05$	$p<0.01$	$p<0.001$
Non-transgenic (ChR2V-)	Contralateral	16-whisker	Tangential	4	0	0	0
			Coronal	4	0	0	0
Transgenic (ChR2V+)	Contralateral	16-whisker	Tangential	4	4	3	1
			Coronal	4	3	3	2
		Single D3	Tangential	4	3	3	3
			Coronal	4	2	2	2
	Ipsilateral	16-whisker	Tangential	2	0	0	0
			Coronal	4	1	1	1
